# Validation of the Visia^®^ Camera System for skin analysis through assessment of the correlations among the three offered measurements – the percentile, feature count and absolute score – as well as the three capture perspectives, from the left, front and right

**DOI:** 10.3205/iprs000165

**Published:** 2022-05-31

**Authors:** Helga Henseler

**Affiliations:** 1 Klinik am Rhein, Klinik für Plastische und Ästhetische Chirurgie, Düsseldorf, Germany

**Keywords:** Visia® Camera System, validation, correlation, percentile, feature count, absolute score

## Abstract

**Objective:**

Validation of the Visia^®^ Camera System in terms of providing data on various skin aspects via the establishment of the correlations among the obtained measurements – the percentile, feature count and absolute score.

**Method:**

A large data cloud was analysed statistically following a clinical study. In the study, facial images of nineteen women were obtained at two different time points, before and three months after following a skin care routine. Objective analysis was provided by the Visia^®^ Camera System, which provided measurements firstly as percentiles, secondly as feature counts and thirdly as absolute scores on eight different skin aspects. The eight skin criteria were spots, wrinkles, skin texture, pores, UV spots, brown spots, red marks and porphyrins. Data on the facial skin were gathered from three different perspectives, namely the left, front and right views. The correlations between pairs of the three obtained measurements, i.e., the percentile, the feature count and the absolute score, were calculated. Further, the correlation coefficients for the three capture perspectives, from the left, front and right, were calculated. Data from the two time points, i.e., before and after application of the skin care cosmetic line were analysed. The statistical analyses were conducted using R (R Core Team 2016).

**Results:**

There was a high level of correlation among the three offered measurement methods. From 144 calculations of the correlations 128 (88.9%) were statistically significant (p<0.05). The correlation coefficients in the vast majority of cases pointed to very clear correlations between the two examined variables. In particular, 50% of the absolute values of these correlations were above 0.945. The few insignificant results were in UV spots and wrinkles. All three methods used to measure the data on skin aspects, i.e., the percentiles, the feature count and the absolute score, served equally well when making comparisons between the two time points. When examining the correlation coefficients for the three capture perspectives, i.e., left, front and right views, their percentages of significant results were found to be only marginally different. Of the 144 examined correlations, 121 were found to be statistically significant (84%). The average correlation coefficient was r=0.74, which pointed to a very clear correlation between the data. The few insignificant results were in wrinkles, UV spots and spots. The Visia^®^ Camera System was found to be an objective tool with which to examine the effects of a cosmeceutical skin care regime. However, there was a learning curve associated with the application of this system.

**Conclusion:**

The Visia^®^ Camera System was successfully validated by investigation of the correlations between measurement methods and capture perspectives. The camera system can not only serve as a tool with which to visualise, provide communications concerning or sell a skin care product, but it can also provide objective data for clinical follow-up studies. Thus, investigations into which skin aspect can be improved the most by a cosmetic product line are possible.

## Zusammenfassung

**Einleitung:**

Validierung der Visia^®^ Kamera in der Datenanalyse zu verschiedenen Hauteigenschaften durch Bestimmung der Korrelationen zwischen den erhobenen Messgrößen – Perzentile, Merkmal in Zahlen und absoluter Wert.

**Methode:**

Eine umfangreiche Datenwolke nach Durchführung einer klinischen Studie wurde statistisch analysiert. In der Studie wurden Bildaufnahmen der Gesichter von neunzehn Frauen zu zwei verschiedenen Zeitpunkten – vor und drei Monate nach Anwendung einer Hautpflegeserie – erhoben. Eine objektive Analyse erfolgte durch die Visia^®^ Kamera, die Messungen erstens als Perzentile, zweitens als Merkmal in Zahlen und drittens als absoluten Wert zu acht verschiedenen Hauteigenschaften anbot. Die acht Hauteigenschaften waren Flecken, Falten, Ebenheit, Poren, UV- Flecken, braune Flecken, rote Bereiche und Porphyrine. Insgesamt wurden Daten der Gesichtshaut aus drei verschiedenen Perspektiven – von links, vorne und rechts – aufgenommen.Die Korrelationen zwischen den angegebenen Messungen – der Perzentilen, dem Merkmal in Zahlen und dem absoluten Wert – wurden berechnet. Es wurden außerdem die Korrelationskoeffizienten zwischen den drei photographischen Perspektiven von links, vorne und rechts kalkuliert. Daten zwischen den zwei Aufnahmezeitpunkten – vor und nach Anwendung der kosmetischen Hautpflegelinie – wurden ausgewertet. Die statistische Analyse erfolgte mit dem Programm R (R Core Team 2016).

**Ergebnisse:**

Es bestand ein hohes Niveau der Korrelationen zwischen den drei angebotenen Messmethoden. Von den 144 Korrelationsberechnungen waren 128 (entspricht 88,9%) statistisch signifikant (p<0.05). Die Korrelationskoeffizienten lagen zum allergrößten Teil in einem Bereich, der auf einen sehr deutlichen Zusammenhang zwischen den je 2 untersuchten Variablen hindeutete. Insbesondere lagen 50% der Absolutwerte der Korrelationskoeffizienten oberhalb eines Wertes von 0,945. Die wenigen nicht statistisch signifikanten Zusammenhänge beschränkten sich auf UV-Flecken und Falten. Alle von der Visia^®^ Kamera angebotenen drei Messmethoden zur Darstellung von Hautaspekten – die Perzentile, das Merkmal in Zahlen und der absolute Wert – dienten gleich gut im Rahmen eines möglichen Vergleiches zu zwei verschiedenen Zeitpunkten. Bei der Untersuchung der Korrelationenskoeffizienten der drei Fotoperspektiven, also links, frontal und rechts, ergaben sich Prozentwerte von signifikanten Ergebnissen, die nur grenzwertig unterschiedlich waren. Von den 144 untersuchten Korrelationen waren 121 statistisch signifikant (84%). Die wenigen nicht signifikanten Ergebnisse fanden sich bei Falten, UV-Flecken und Flecken. Die Visia^®^ Kamera diente als objektives Werkzeug um die Effekte einer Hautpflegeserie nachzuvollziehen. Allerdings bestand eine Lernkurve bei der Anwendung der Visia^®^ Kamera.

**Schlussfolgerung:**

Die Visia^®^ Kamera wurde durch Untersuchung der Korrelationen zwischen verschiedenen Messmethoden und Aufnahmeperspektiven erfolgreich validiert. Die Kamera kann nicht nur als Werkzeug dienen, um ein Hautpflegeprodukt darzustellen, zu erläutern oder zu verkaufen, sondern kann ebenfalls objektive Daten im Rahmen einer klinischen Follow-up-Studie zur Verfügung stellen. Die Möglichkeit zu analysieren, welcher Hautaspekt am besten durch eine kosmetische Produktlinie verbessert werden kann, wird eröffnet.

## Introduction

Modern digital imaging methods have emerged in the last decades and continue to develop with advancements in computer science [1]. Using a new method of data capture and analysis, several aspects of digital imaging can be examined to assess the quality of the capture system [2]. The aspects available for study depend on the system in use. The Visia^®^ Camera System from Canfield Scientific, Inc., New Jersey, USA (https://www.canfieldsci.com/) is considered to be the most sensitive camera system on the market [3]. The Visia^®^ Camera relies on three different measurements, namely the percentile, the feature count and the absolute score. The percentile provides a comparison of a person’s complexion to those of people with similar skin characteristics. Percentiles can be used when a baseline assessment of the overall condition of a complexion is desired. The feature count totals discrete instances of the feature of interest without regard for size or intensity and is used to track treatment progress when just the development number-wise of a skin criterium is examined. The absolute score offers a comprehensive measurement of a skin criterium of interest as it captures total size and area as well as intensity. Thus, this score, which is also called the value in some instances, can serve to track the progress of a treatment over time with regard to these criteria. All three of these methods are applied in the analysis of the captured data. In principle, the camera examines eight different skin aspects. Due to the use of three measurement methods, the first question that might arise is whether all three methods contribute equally to the results and therefore whether correlations among the three measurement methods might exist. The examination of these correlations provides valuable data on the relations among these measurements [4], [5], [6]. The camera system also offers the option of capturing the face from the left, front and right views. Using each of these three perspectives, eight skin aspects are measured. Therefore, the question arises as to whether all three perspectives contribute equally to the overall skin analysis. Additionally, facial captures can be conducted at different time points, so it would be interesting to learn whether correlations exist between captures from different time points. Hence, in a validation of a digital imaging system, many different features can be examined to provide an overall picture regarding the quality of the system. 

## Aim

To determine whether the three measurement methods provided by the Visia^®^ Camera system correlate to each other and can perform equally well in providing skin analyses; and further to assess the correlations between capture perspectives and different time points.

## Methods

**Figure 1:**
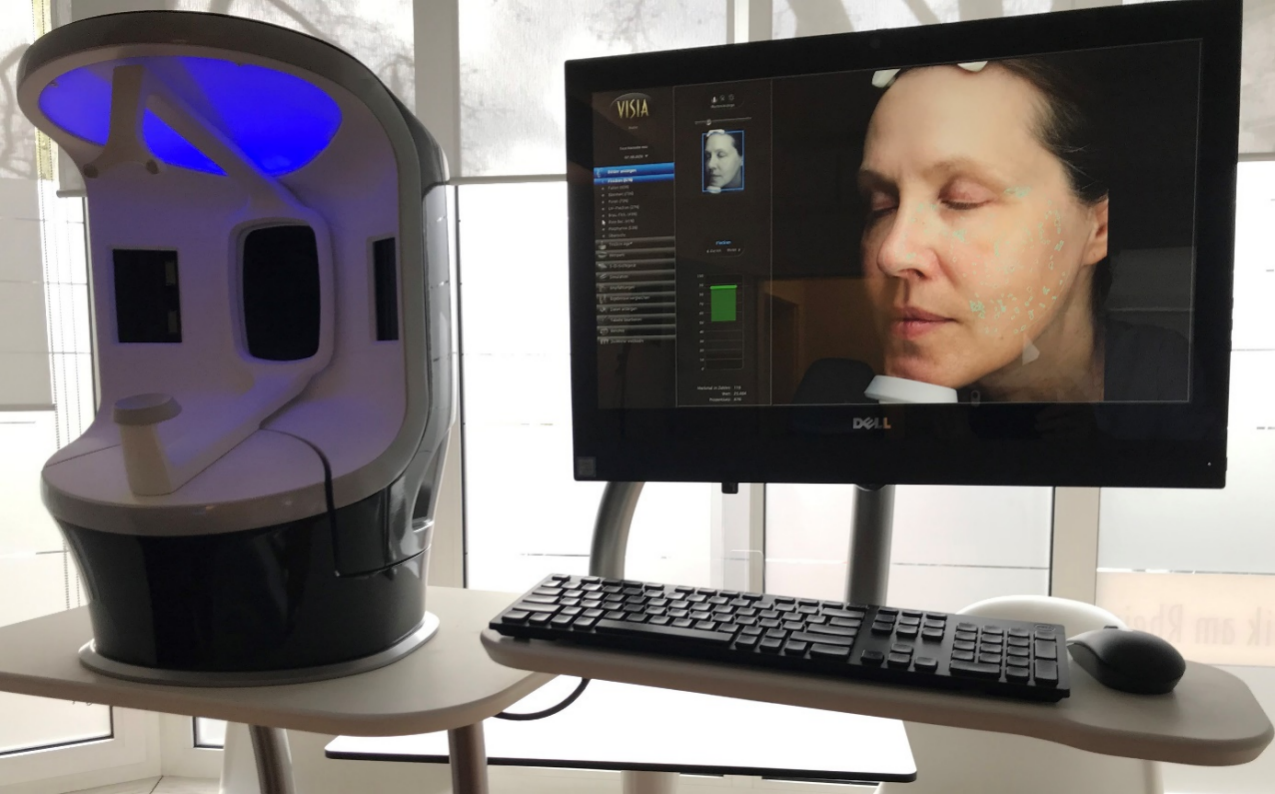
The Visia^®^ Camera System for complexion analyses

The study firstly collected data on all the variables of interest on facial images using the Visia^®^ Camera System provided by Canfield Scientific (Figure 1 (Fig. 1)). Via the application of several flashes, numerous skin features were displayed. Eight different skin surface aspects were examined. The eight skin criteria were spots, wrinkles, skin texture, pores, UV spots, brown spots, red marks and porphyrins.

Firstly, the correlation coefficients among the three measurements, i.e. the percentile, the feature count and the absolute score, were calculated for each of the eight skin criteria, then separately for the three capture perspectives as well as both time points. For each criterium, three calculations of the Spearman correlation were carried out: firstly, the Spearman correlation for percentile and feature count was found; secondly, the correlation between the percentile and absolute score was calculated; and thirdly, the correlation between the feature count and absolute score was computed. 

Secondly, the correlation coefficients among the three capture perspectives, i.e., left, front and right view, were found for the obtained measurements, i.e., percentile, feature count and absolute score, for each of the eight criteria and separately for both time points. 

The program R was used for the statistical calculations (R Core Team 2016). R (https://www.R-project.org) is a language and environment for statistical computing developed by the R Foundation for Statistical Computing, Wien [7]. All images were created with the packet “ggplot2” in R. 

All variables were interval-scaled as well as continuous. Thus, for each variable, the calculations included the amount of valid data as well as the mean, standard deviation, median, minimum and maximum. Results were considered statistically significant at a p-value less than 0.05 [5]. The p-value was calculated with the Wilcoxon test to establish statistical significance.

The Spearman correlation coefficient r measures the strength of the relationship between two arbitrarily distributed, continuous variables. The correlation can assume values between –1 and +1. The closer r is to –1 or 1, the stronger the correlation is between the examined variables. When r is positive, the correlation is direct, which means that as one variable increases, the other increases too. If r is negative, the correlation is indirect; in this case, if the one variable increases, the other decreases. Measurements of r larger than 0,71 point to a very clear correlation between variables.

Data were recorded as “percentile”, “feature count” and “absolute score” by the Visia^®^ Camera System. A rise value indicated an improvement in the percentile whereas a fall in value pointed to an improvement in the feature count as well as the absolute score.

## Results

### The correlations for the measurement methods

The correlation coefficients for the three measurement methods were calculated for each of the eight skin criteria, then separately for the three capture perspectives as well as both time points, resulting in 144 correlations (3x8x3x2). Of these correlations, 128 (88.9%) were statistically significant (p<0.05). 

When examining the correlation results for the three capture perspectives, i.e., left, front and right views, their percentages of significant results were found to be only marginally different. For the left perspective, 41 out of 48 correlations (85.4%) were found to be statistically significant; from the front perspective, 44 correlations (91.7%) were statistically significant; and from the right perspective, 43 correlations (89.6%) were statistically significant. Thus, the correlation coefficients in the vast majority of cases pointed to very clear correlations between the two examined variables. In particular, 50% of the absolute values of these correlations were above 0.945. 

The remaining correlations that were not statistically significant were limited to two criteria: UV spots and wrinkles. For UV spots, only six out of 18 correlations (33.3%) were statistically significant, whereas for wrinkles, 14 out of 18 correlations (77.8%) were statistically significant. Thus, for these criteria, the data collection methods should perhaps be regarded as less reliable than those for the other criteria. 

**Figure 2:**
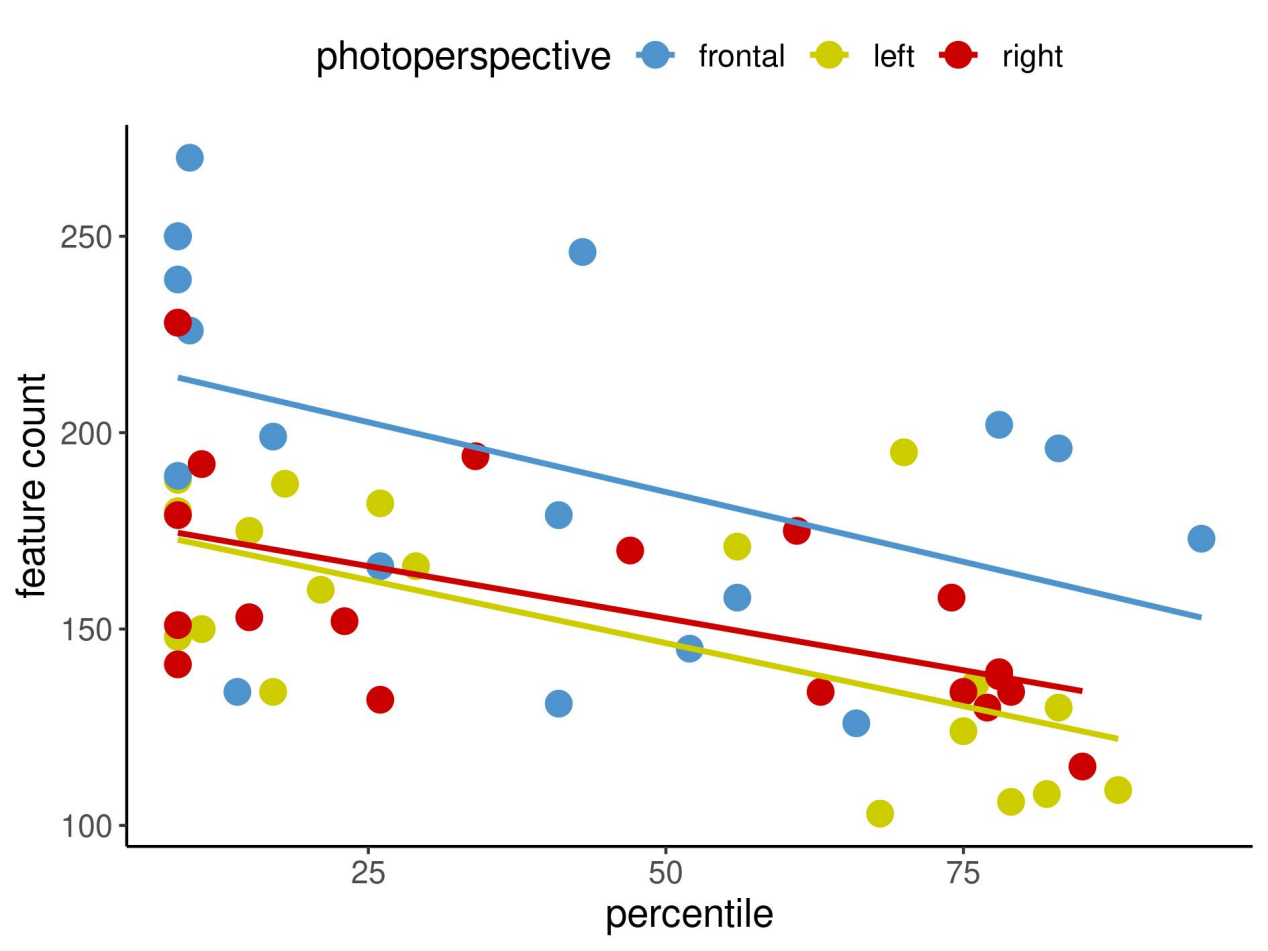
Correlations between percentile and feature count for SPOTS for the three capture perspectives at time point 1

**Figure 3:**
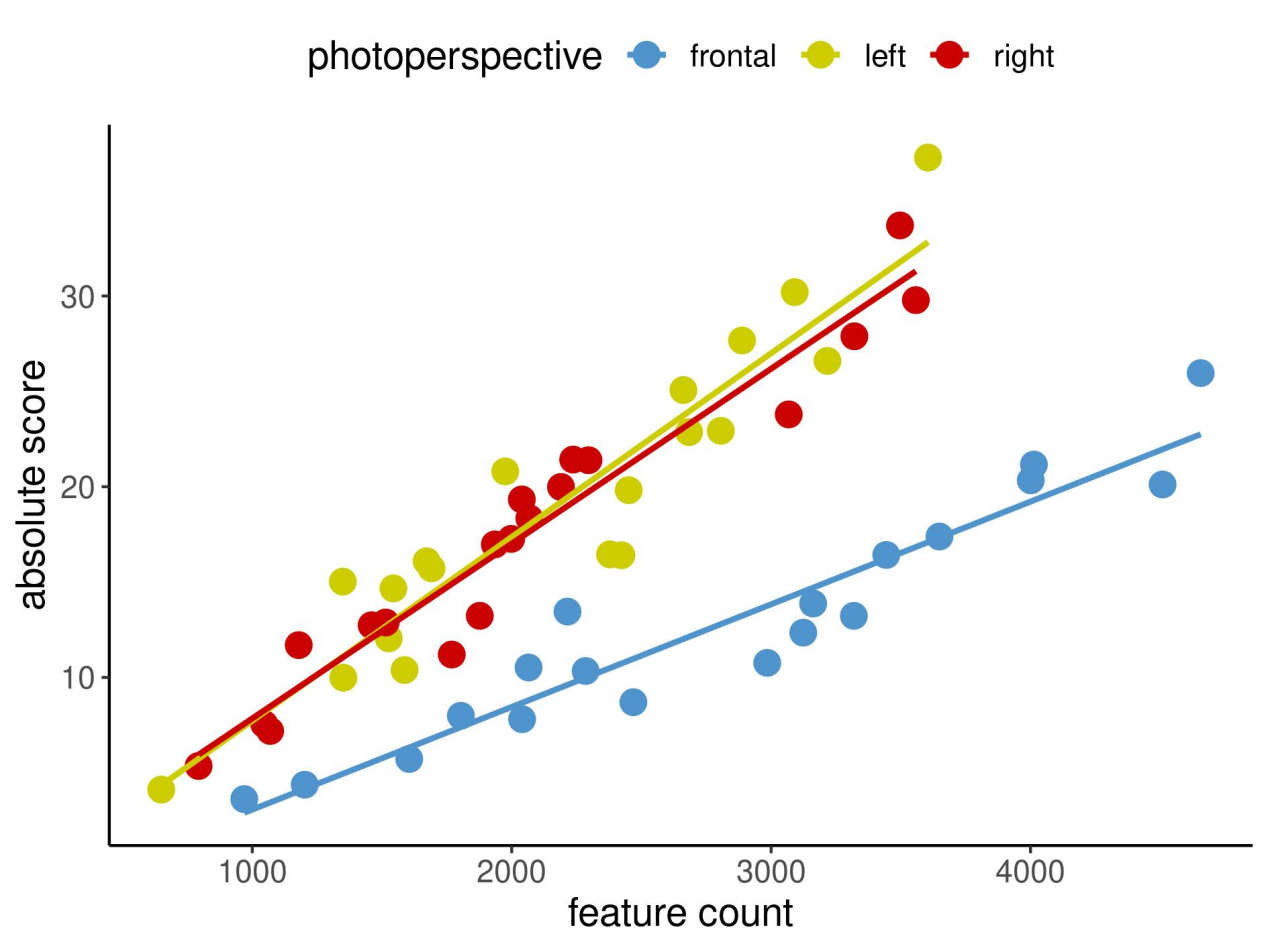
Correlations between feature count and absolute score for TEXTURE for the three capture perspectives at time point 1

**Figure 4:**
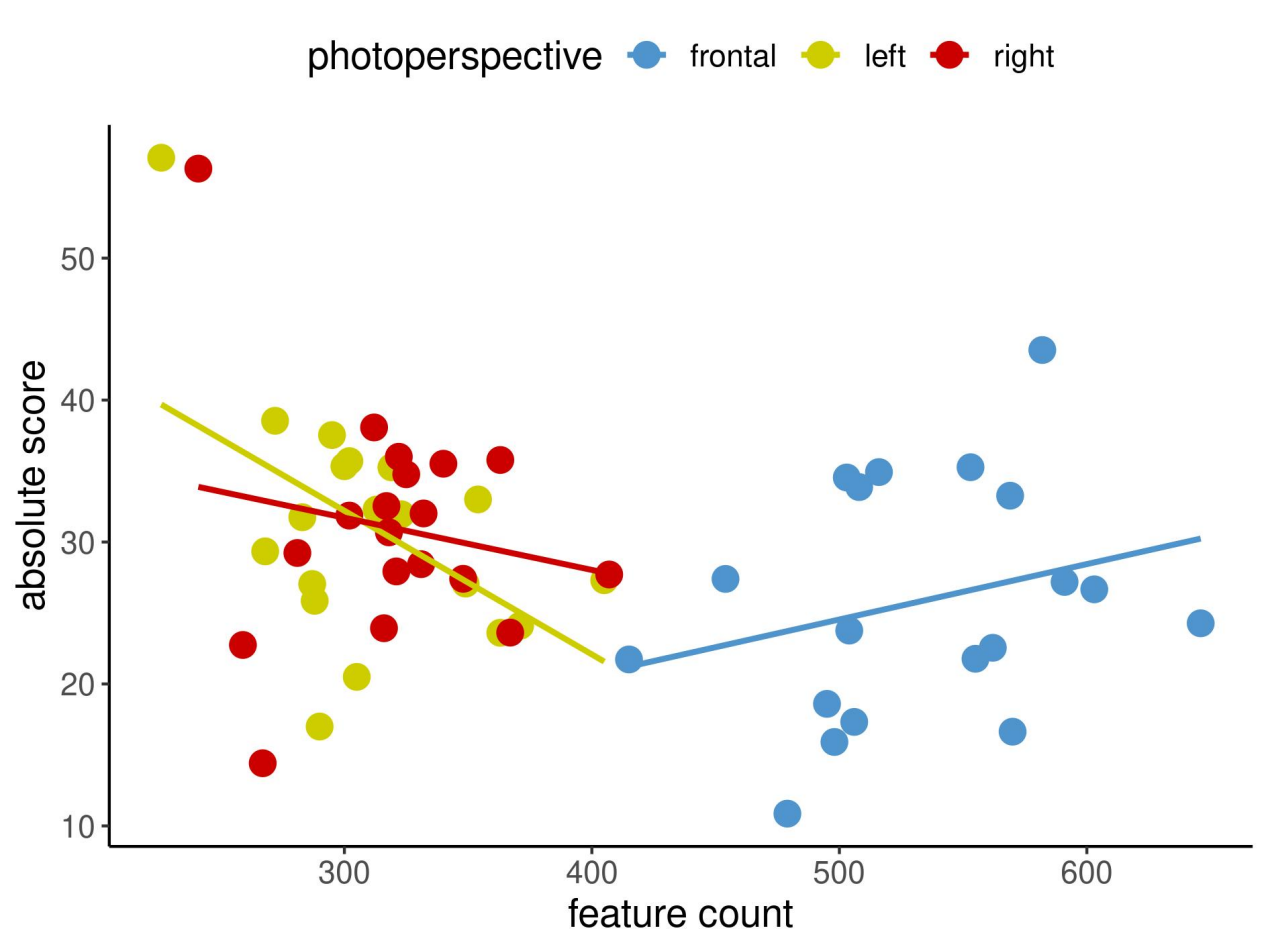
Correlations between feature count and absolute score for UV SPOTS for the three capture perspectives at time point 2

Figure 2 (Fig. 2), Figure 3 (Fig. 3) and Figure 4 (Fig. 4) show in an exemplary way the correlations among the measurement methods for spots (Figure 2 (Fig. 2)) and texture (Figure 3 (Fig. 3)) at the first time point as well as UV spots (Figure 4 (Fig. 4)) at the second time point. In each image, the three different capture perspectives, namely front, left and right, are displayed in different colours. It appears that the measurement data for spots exhibits weaker correlations than those for texture. However, the data for UV spots vary the most, resulting in the weakest correlations between the measurements. 

### The correlations for the capture perspectives

**Figure 5:**
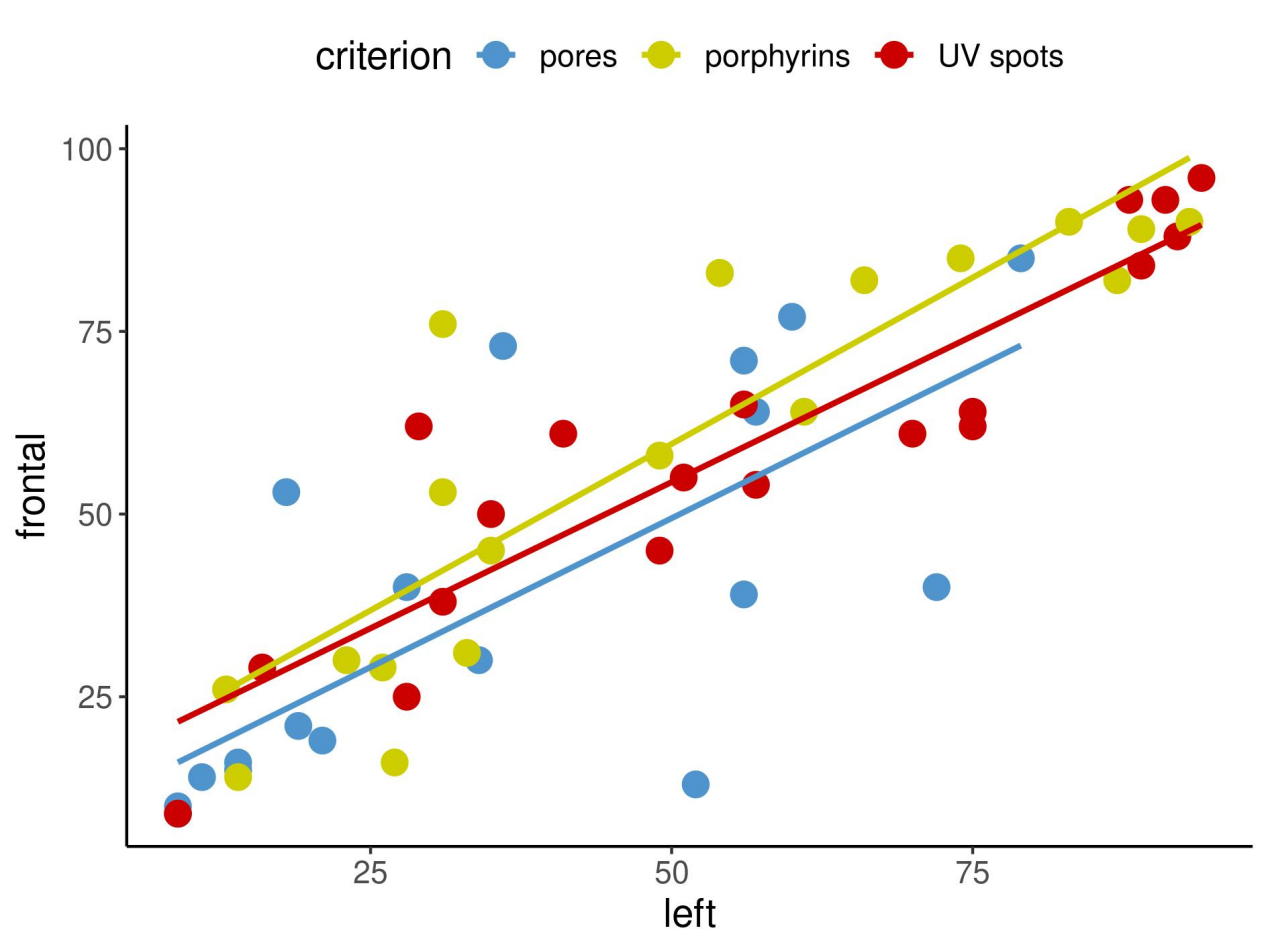
Correlations between percentile left and percentile front for pores, porphyrins and UV spots at time point 1

**Figure 6:**
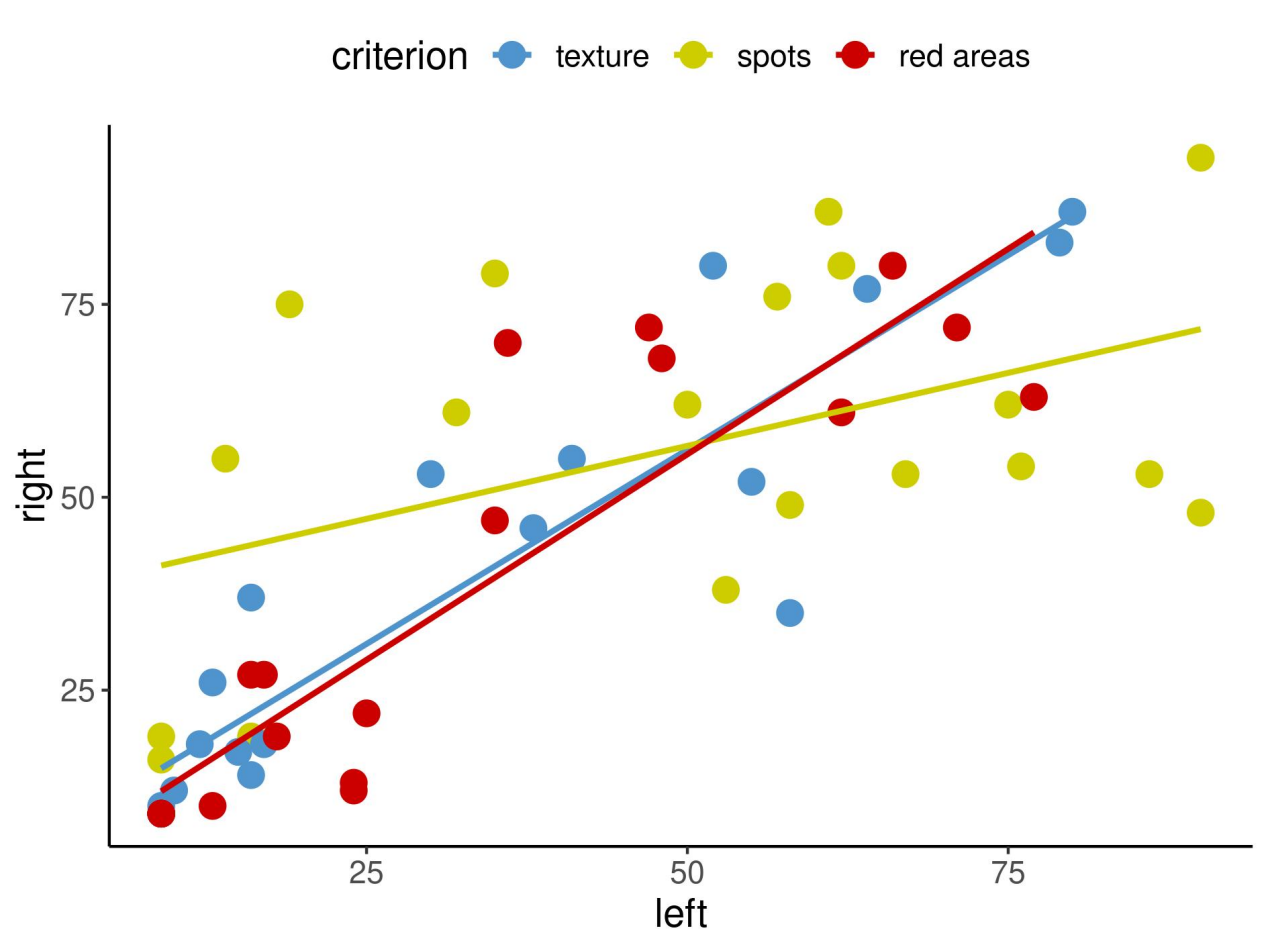
Correlations between percentile left and percentile right for texture, spots and red areas at time point 2

Secondly, correlation coefficients among the three capture perspectives, namely left, front and right, were calculated, and some of the results are displayed in Figure 5 (Fig. 5) and Figure 6 (Fig. 6). The calculations of the paired correlations were done between the left and front, left and right and front and right views for each of the eight criteria at both time points. 

Again, 144 correlations were assessed: three measurements of eight criteria across two timepoints and three capture perspectives. 

Of the 144 examined correlations, 121 were found to be statistically significant (84%). The average correlation coefficient was r= 0.74. This pointed to a very clear correlation between the data.

Of the 23 correlations that were not statistically significant, 13 involved the criteria wrinkles, five involved UV spots and five involved spots. In seven cases, the correlations among the three capture perspectives for percentile and feature count were classified as not statistically significant whereas additional nine cases involved absolute score. 

The correlations between left and frontal perspective revealed that there were nine results that were not statistically significant. For the correlations between the left and right views, there were four cases in which the results were not statistically significant. For the correlations between frontal and right views, there were 10 results that were not statistically significant.

Figure 5 (Fig. 5) and Figure 6 (Fig. 6) show in an exemplary way the correlations for the capture perspectives for pores, porphyrins and UV spots at timepoint 1 (Figure 5 (Fig. 5)) and for texture, spots and red areas at timepoint 2 (Figure 6 (Fig. 6)). 

## Discussion

The results demonstrated that there were relatively few correlations that were not statistically significant. The question then arises as to what this means for the imaging system that was examined and what conclusions can be taken regarding these findings for clinical practice. An insignificant result merely indicates that no firm conclusion can be made regarding the assessed correlation [8]. Importantly, 89% of the examined correlations were significant, revealing a very clear tendency: high values for one measurement method in a specified view tended to be accompanied by high values for the other measurement method being considered, as displayed in Figure 2 (Fig. 2), Figure 3 (Fig. 3) and Figure 4 (Fig. 4). The same was true for low values. Figure 2 (Fig. 2), Figure 3 (Fig. 3) and Figure 4 (Fig. 4) cover three examples of skin criteria, for which a total of 144 correlations were examined. The same was true when assessing the correlations among the three capture perspectives, namely the left, front and right views, as displayed in Figure 5 (Fig. 5) and Figure 6 (Fig. 6). In the vast majority of cases (84%), a clear tendency was revealed in which results for one capture perspective went along with those for the other perspectives. Therefore, when examining the results, it is not important to understand why some results were not statistically significant but rather to realise that in the overall majority of cases, a clear correlation was found. Based on these significant correlations, it can therefore be concluded that the capture system presented reliable data in the comparison of the three different measurement methods, i.e., percentile, feature count and absolute score, and the three capture perspectives, which can all objectively assess skin surface features. 

The Visia^®^ Camera System’s percentile measurements most clearly presented the results to the user, which was evident in both the initial capture and the overview of the images. The individual percentile results are presented in relation to a group of people of same age and skin colour and comparisons are visualised with green and red bars when indicating whether an individual has more or less, respectively, of the skin criterium of interest. Interestingly, the results for the other two measurement methods, feature count and absolute score, are presented in less obvious ways by the camera system, and the user is left confused as to which measurement to use when all three measurements are provided next to each other. 

Overall, the absolute score provides the most detailed data cloud for the skin criterium, making it the most comprehensive measurement method. Therefore, this measurement method is recommended to be used when comparing measurements from two timepoints, such as before and after a treatment, to assess skin quality precisely and longitudinally. Nevertheless, the results of this examination reveal that the two other measurement methods, namely percentile and feature count, can also be trusted to provide objective skin surface assessments since all three measurement methods relate to each other. When the question comes up as to which of the three measurement methods available for the Visia^®^ Camera System should be used, the answer is that it depends on the context. In an initial skin analysis, the percentile is fine; whereas for research questions, the absolute score is preferable. The feature count is helpful for communication purposes as it provides easy to understand information to the discrete number of a skin feature of interest. There is a learning curve associated with the application of this system. 

One of the findings was that there were some insignificant results. It could be postulated that these insignificant results might have been fewer in number if the data cloud had been larger. An investigation of this question however was beyond the scope of this research paper. Nevertheless, altogether the correlations between the variables were very clear, which speaks to the reliability of the capture system. 

In the literature on validation methods for objective digital capture systems, frequently just a single feature, such as surface area, is examined [9]. Therefore, data comparison remains a challenge. One application of the Visia^®^ Camera System to date for example was an analysis of the single feature of acne scarring [10], [11], for which the system was judged a suitable tool for analysis. In contrast, this study here examined numerous skin surface aspects. With this study numerous features were independently examined with different measurement methods and capture perspectives at different time points. Interestingly, this study determined that especially for UV spots, but also for wrinkles, there were weaker correlations among the measurement methods and capture perspectives. Hence, for these criteria, the Visia^®^ Camera System results should be taken with care. Additional measurement methods might need to be taken into consideration for these features. Validation studies remain time-consuming and costly, and results are often not as clear as desired [12], [13]. Thorough, independent validation efforts however remain important and desirable. In this study the validation of the system was pursued contributing to the small number of publications on this matter. 

## Conclusion

The Visia^®^ Camera System was successfully validated by investigation of the correlations between measurement methods and capture perspectives. The camera system can not only serve as a tool with which to visualise, provide communications concerning or sell a skin care product, but it can also provide objective data for clinical follow-up studies, Thus, investigations into which skin aspect can be improved the most by a cosmetic product line are possible. 

## Notes

### Acknowledgment

The author thanks Dr. Wolfgang Reimers for his support in the statistical analysis of the data. 

### Ethical statement

All procedures performed in the study were done in accordance with the ethical standards of the institutional and national research committee and with the 1964 Helsinki declaration and its later amendments and comparable ethical standards.

### Competing interests

The author declares that she has no competing interests. This was an independent investigation of the Visia^®^ Camera System.
